# Liquid Biopsy: A Biomarker-Driven Tool towards Precision Oncology

**DOI:** 10.3390/jcm9082556

**Published:** 2020-08-07

**Authors:** Nelson S. Yee

**Affiliations:** 1 Division of Hematology-Oncology, Department of Medicine, Penn State Health Milton S. Hershey Medical Center, Hershey, PA 17033-0850, USA; nyee@pennstatehealth.psu.edu; Tel.: +1-717-531-5868; 2 Next-Generation Therapies Program, Penn State Cancer Institute, Hershey, PA 17033-0850, USA; 3 The Pennsylvania State University College of Medicine, Hershey, PA 17033-0850, USA

## 1. Liquid Biopsy towards Precision Oncology

Liquid biopsy or the sampling of bodily fluids, mostly blood, has been intensely investigated and developed for clinical utility in medicine, especially oncology [1]. Analysis of the cellular and/or molecular contents of the blood-based biopsy has been demonstrated to yield predictive biomarkers that help decisions on anti-cancer targeted therapy and monitor tumor response to treatment. Emerging evidence indicates the potential of plasma-derived molecules for early detection and diagnosis of various malignant diseases. Besides, research efforts have been focused on the methodologies in an attempt to improve the efficiency of isolation and analysis of biomarkers collected from blood-based biopsies. The ultimate goal is to develop an accurate, rapid, and cost-effective technology of liquid biopsy that can be used for a point of care in the clinical practice of precision oncology.

In this article [2], Mathai and others reported a comprehensive review of liquid biopsy as well as their application for diagnosis and prognostication in some of the most common solid tumors. Various aspects of liquid biopsy, including its advantages and disadvantages, are compared with the conventional surgical biopsies. The three major types of biomarkers being isolated and analyzed in liquid biopsy, including circulating tumor DNA (ctDNA), circulating tumor cells (CTCs), and exosomes, are discussed. The methodologies for isolation and analysis of liquid biopsy are presented. These include commercially available kits for the isolation of cell free DNA (cfDNA), the Food and Drug Administration (FDA) approved instrument CELLSEARCH^®^ for capturing and enumerating CTCs, and conventional methods for the isolation of exosomes using Western blotting and enzyme linked immunosorbent assay (ELISA). Analysis of the genetic contents in the liquid biopsied biomarkers by advanced techniques of polymerase chain reactions (PCR) and next-generation technology is described. The clinical utility of liquid biopsy in colorectal, breast, hepatocellular, gastric, and lung carcinomas is discussed. By enabling the detection of genetic alterations in the tumors, liquid biopsy-based biomarkers may be utilized for predicting tumor response to treatment, monitoring anti-tumor response during therapy, and determining prognosis. 

## 2. Liquid Biopsy in Pancreatic Cancer

While pancreatic cancer (PC) is relatively uncommon, its mortality rate has remained the highest among all malignant diseases [3]. PC is frequently diagnosed at an advanced stage, such that palliative chemotherapy with supportive care is the only treatment option [4]. Early detection and diagnosis of PC is of the utmost importance for multi-disciplinary treatment with curative intent [5]. Conventional imaging modalities with tissue biopsy are able to diagnose symptomatic PC [5,6,7], though relatively late in the disease course, except when they are performed for seemingly unrelated causes and a pancreatic mass is incidentally identified. In recent years, liquid, particularly blood-based biopsy, has emerged and offered a new opportunity for the early detection and diagnosis of PC.

As demonstrated in a number of studies, CTCs, ctDNA, and extracellular vesicles (including exosomes and microvesicles) have been detected in patients with PC [8,9,10]. Analysis of plasma-derived ctDNA as well as the molecular cargoes of CTCs and EVs has yielded a variety of cancer-related biomarkers [8,9,10]. Preliminary studies have demonstrated the potential of a combination of these blood-based biomarkers for early detection of PC [11,12,13,14]. A prospective study shows the correlation of exosomal DNA and ctDNA in blood-based biopsies with clinical outcomes and suggests the utility of these nucleic acids for therapeutic stratification [15]. A meta-analysis indicates a relatively high sensitivity, specificity, and accuracy of liquid biopsy, which can serve as a surrogate for tissue in the molecular profiling of PC [16]. With the hope of developing liquid biopsy for clinical utility, various technological approaches have been focused on optimizing the isolation of blood-based biomarkers from patients with PC. 

## 3. Conclusions and Future Perspectives

**Figure 1:**
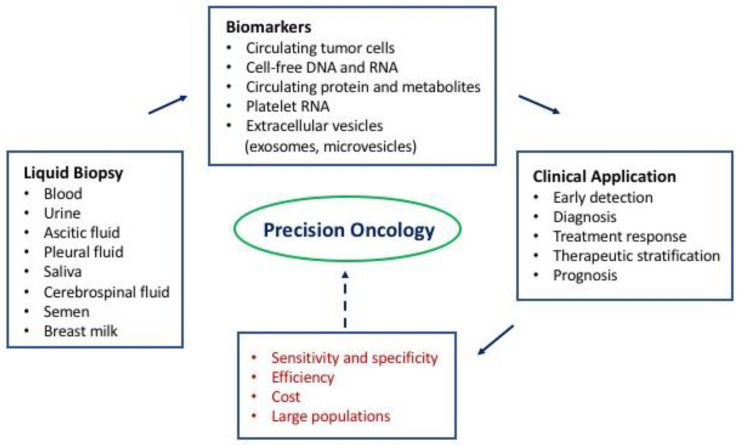
A schematic diagram for liquid biopsy towards precision oncology. Liquid biopsy enables the isolation and identification of molecular biomarkers that facilitate early detection and diagnosis of cancer, as well as monitoring treatment response, stratification of patients for therapy, and assessing prognosis. Further investigation of liquid biopsy is indicated for attaining the goal of precision oncology by (i) improving the sensitivity and specificity of the biomarkers for various clinical applications in patients with cancer, (ii) improving the efficiency and cost of the technologies for isolation and analysis of the biomarkers, and (iii) validating the accuracy of the biomarkers in prospective studies with large populations.

Accumulating evidence indicates the clinical utility of liquid biopsy for potential biomarkers in patients with malignant diseases (Figure 1). The combined features of liquid biopsy including minimal invasiveness, repeatability, and relatively low cost have enabled it to emerge as a biomarker-driven tool complementary to biopsy of tumor tissues. Continued research efforts to improve the accuracy, efficiency, and cost effectiveness of liquid biopsy as well as to validate its clinical utility in prospective studies with a large number of patients from various geographical locations are indicated to attain the goal of precision oncology.
